# Multiyear Maize Management Dataset collected in Chiapas, Mexico

**DOI:** 10.1016/j.dib.2022.107837

**Published:** 2022-01-17

**Authors:** Rodrigo G. Trevisan, Nicolas F. Martin, Simon Fonteyne, Nele Verhulst, Hugo A. Dorado Betancourt, Daniel Jimenez, Andrea Gardeazabal

**Affiliations:** a Department of Crop Sciences, University of Illinois at Urbana-Champaign, 1102 Goodwin Ave. Urbana, IL, USA; b International Maize and Wheat Improvement Center (CIMMYT), Carretera México-Veracruz Km. 45, El Batán, Texcoco, Mexico; c International Center for Tropical Agriculture (CIAT) Km 17 Recta Cali-Palmira, 763537 - A.A. 6713, Cali, Colombia; d Universidad Icesi. Calle 18 # 122-135, Valle del Cauca 760031, Pance, Cali, Colombia

**Keywords:** Explanatory machine learning, Sustainable intensification, Smallholders, Tropical agriculture

## Abstract

For several decades, maize (*Zea mays* L.) management decisions in smallholder farming in tropical regions have been a puzzle. To best balance alternative management practices' environmental and economic outcomes, an extensive dataset was gathered through CIMMYT's knowledge hub in Chiapas, a state in southern Mexico. In a knowledge hub, farmers, with the support of farm advisors, compare conventional and improved agronomic practices side-by-side and install demonstration fields where they implement improved practices. In all these fields data on on-farm operations and results is collected. The dataset was assembled using field variables (yield, cultivars, fertilization and tillage practice), as well as environment variables from soil mapping (slope, elevation, soil texture, pH and organic matter concentration) and gridded weather datasets (precipitation, temperature, radiation and evapotranspiration). The dataset contains observations from 4585 fields and comprises a period of 7 years between 2012 and 2018. This dataset will facilitate analytical approaches to represent spatial and temporal variability of alternative crop management decisions based on observational data and explain model-generated predictions for maize in Chiapas, Mexico. In addition, this data can serve as an example for similar efforts in Big Data in Agriculture.

## Specifications Table

**Table:** 

Subject	Agronomy and Crop Science
Specific subject area	Smallholder Tropical Maize Farming
Type of data	Table
How the data were acquired	Field management data and grain yield data were collected by farm advisors during regular field visits. Soil data were obtained from Mexico's National Institute of Statistics and Geography, INEGI. Weather data were obtained from Daymet. Municipal yield data were obtained from Mexico's Agri-food and Fisheries Information Service, SIAP.
Data format	Raw data
Description of data collection	Modules and extension areas were installed by farmers together with farm advisors. The farm advisors captured management data in the Bitacora Electronica MasAgro system, through which the data was aggregated. Data were collected for 4585 fields spread over seven growing seasons (2012-2018). For each field weather and soil data were obtained from Daymet and INEGI.
Data source location	• Institution: International Maize and Wheat Improvement Center (CIMMYT) • State: Chiapas • Country: Mexico:
Data accessibility	Repository name: Dataverse Data identification number (Handle): 11529/10548624 Direct URL to data: https://hdl.handle.net/11529/10548624 Instructions for accessing these data: the data are freely available.

## Value of the Data

• This dataset provides an unprecedented amount of information about maize growing conditions in an essential and diverse tropical production region in Mexico.
• The dataset can be used in different types of studies focused on maize management, from an agricultural, germplasm improvement, and environmental perspective.
• Some possible applications of this dataset include comparing different cropping system characteristics and evaluating maize management techniques and agricultural policies.
• Educators can use the dataset for developing machine learning problems, statistics, or data mining training.
• The dataset serves as an example for other crops and regions of the value of collecting standardized farming datasets for public distribution.
• The dataset can be used to test approaches like modeling, machine learning and Big Data mining to improve decision support for smallholder farmers.

## 1 Data Description

The dataset [1] consists of a single table integrated by 4 main types of variables: identification of field, maize yield and field management, soil characteristics, and weather conditions during the season. Each of the 4585 rows in the table corresponds to a unique field for the corresponding season. Table 1 describes each of the variables along with the units of measurement.

**Table 1:** Variables included in the data file, including description and units.

Variable	Description	Unit
**Field_ID**	Unique identifier	
**Lat**	Latitude coordinates	Degrees
**Long**	Longitude coordinates	Degrees
**Elev**	Elevation	m
**Mun_Yield**	Average maize yield in a municipality	Mg ha^−^**^1^**
**System**	Specification of hybrid or landrace-based cropping system	
**Yield**	Field maize yield	Mg ha^−^**^1^**
**Cultivar**	Genotype information	
**Tillage**	Tillage practices including Conventional, Reduced, and No-Till	
**Planting**	Day of the year when maizes was planted	DOY
**Nitrogen**	Nitrogen added as fertilizer	kg N ha^−1^
**Phosphorus**	Phosphorus added as fertilizer	kg P_2_O_5_ ha^−1^
**Potassium**	Potassium added as fertilizer	kg K_2_O ha^−1^
**Slope**	Field slope	%
**Clay**	Soil clay content	%
**CEC**	Cation Exchange Capacity	cmolc dm^−3^
**SOM**	Soil Organic Matter	**%**
**pH**	Soil pH	
**prcp(V1-V30)**	Precipitation per day over 10 days from V1 to V30	mm day^−1^
**srad(V1-V30)**	Solar radiation per day over 10 days from V1 to V30	MJ m^−2^ day^−1^
**tmax(V1-V30)**	Maximum temperature over 10 days from V1 to V30	^o^C
**tmin(V1-V30)**	Minimum temperature over 10 days from V1 to V30	^o^C
**Vp (V1-V30).**	Vapor pressure over 10 days from V1 to V30	Pa

**Fig. 1:**
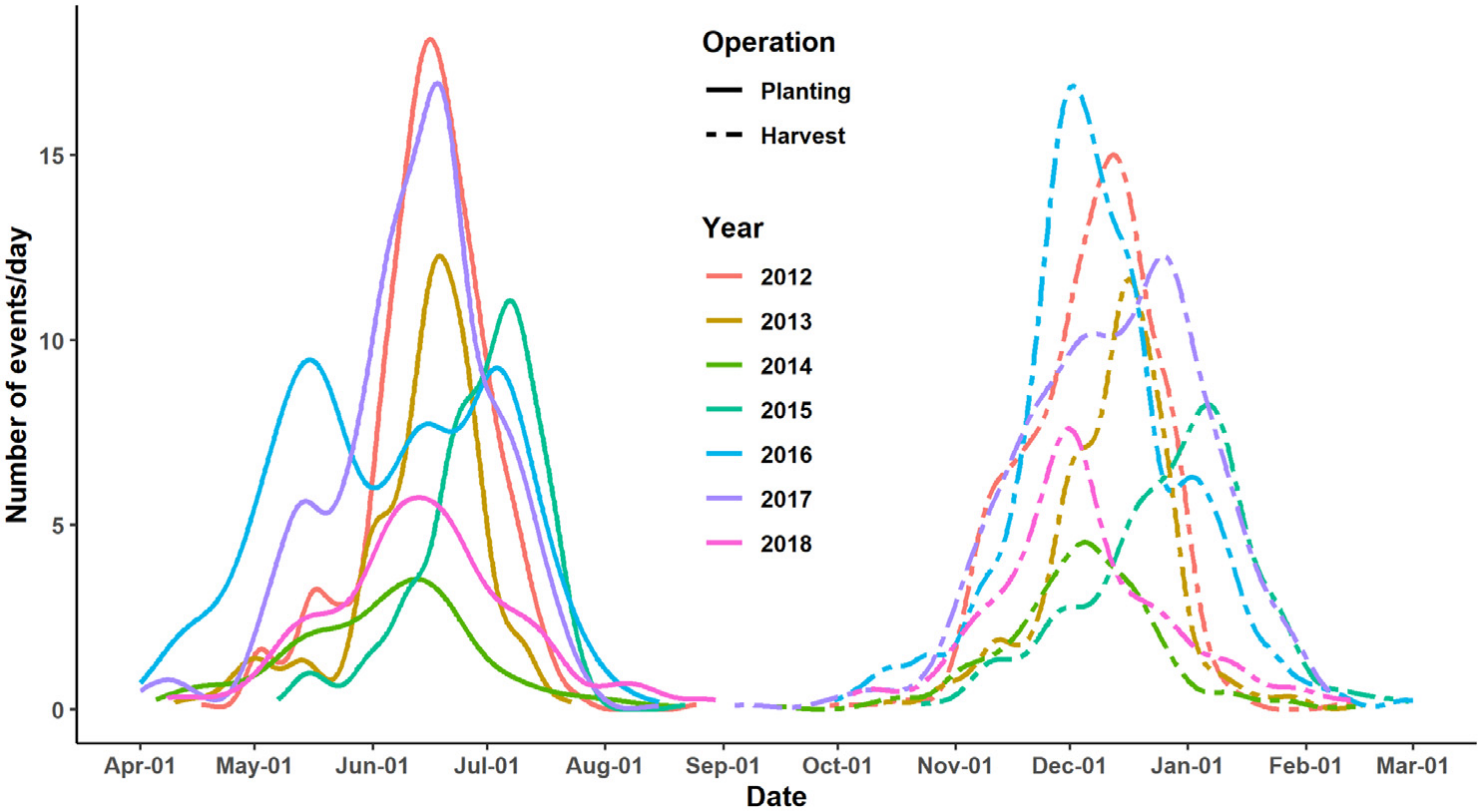
Temporal distribution of planting and harvesting maize cropping events in seven years of field observations from Chiapas – Mexico. Farmers in the region tend to keep maize in the field for long periods after physiological maturity due to a lack of resources for timely manual harvesting. Therefore, the season length is not a good representation of the growing period.

The yield ranged from 0.1 to 10.0 Mg ha^−1^, with 75% of the values lower than 5.0 Mg ha^−1^ and an average of 6 Mg ha^−1^ (Table 2). The elevation ranged from almost sea level to about 3000 m above sea level, although observations were concentrated around the median of 700 m above sea level. The planting dates had a standard deviation of 20 days, with some observations up to five months apart from others. The number of observations recorded in some years varied considerably, with a smaller number in 2014 and increasing later (Fig. 1). The total season had an average duration of 175 days.

**Table 2:** Descriptive statistics of the numerical variables in the dataset of maize cropping events using seven years of field observations from Chiapas, Mexico.

*SD: standard deviation; P0 – P100: data distribution percentiles; CEC: cation exchange capacity; SOM: soil organic matter; DOY: day of the year.

More than 25% of farmers did not use phosphorus fertilizer, while more than 50% did not use potassium fertilizer (Table 2). The average nitrogen rate was 110 kg N ha^−1^, with some farmers applying up to triple this amount. Most zero nitrogen rates were attributed to farmers not reporting the application or applying nitrogen from other sources such as manure.

Conventional tillage was the most common, followed by no-till (Table 3). A total of 250 unique cultivars were recorded, with many of them in low frequency. The number of observations for each cultivar significantly changed between years due to hybrid turn-over.

**Table 3:** Descriptive statistics of the categorical variables in the dataset of maize cropping events using seven years of field observations from Chiapas, Mexico.

Variable	Number of levels	Most frequent
Year	7	2017: 960, 2016: 816, 2018: 807, 2012: 723
System	2	Hybrid: 3034, Landrace: 1551
Cultivar	250	Criollo (unspecified landrace): 629, P4082W: 420
Tillage	3	Conventional: 2092, No-till: 1634, Reduced: 859

**Fig. 2:**
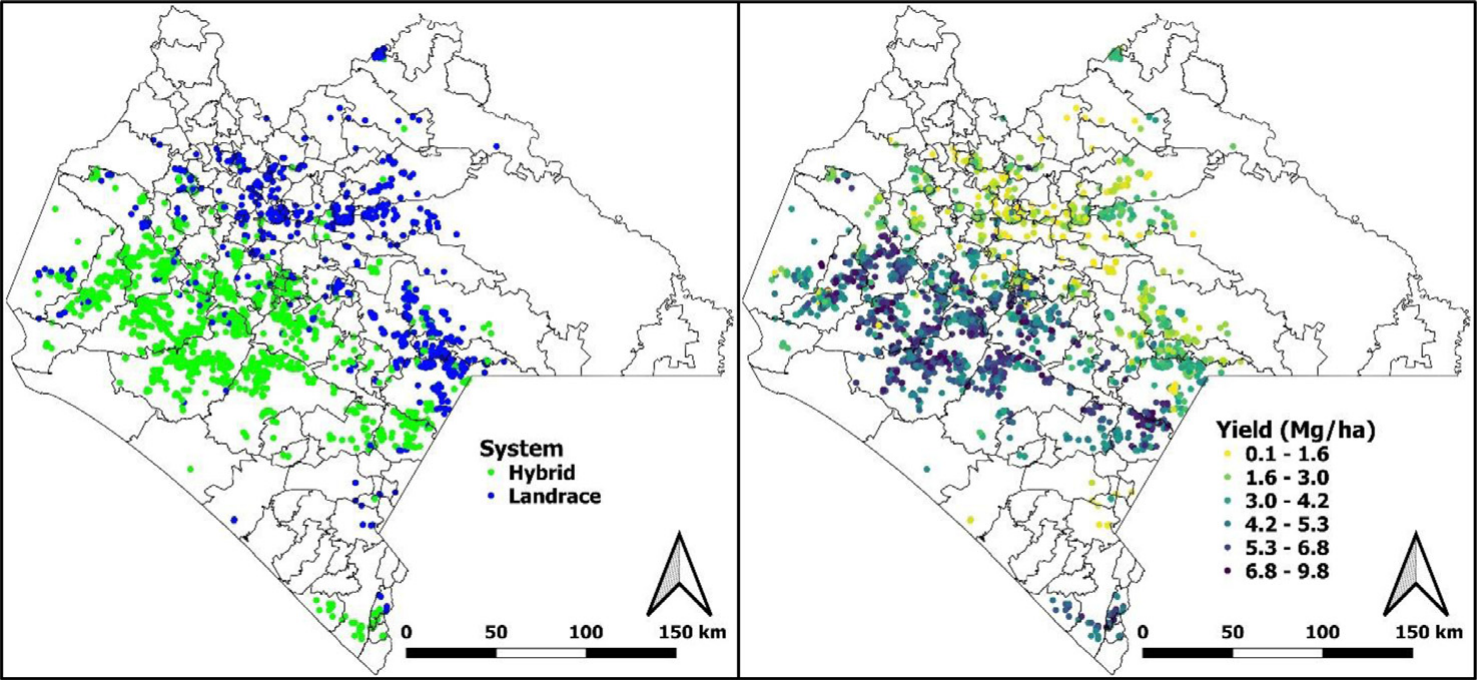
Spatial distribution of system type and maze yield using seven years of field observations from Chiapas, Mexico.

There is a clear separation in the spatial distribution of system type and maize yield (Fig. 2). A Hybrid system characterizes the central west part of the state. The north and east municipalities are characterized by small-scale self-consumption farmers using mainly landraces. The yields follow the same pattern, with most observations in the range of 1.6 to 3.0 Mg ha^−1^ for the Landrace system and 4.2 to 5.3 Mg ha^−1^ in the Hybrid system.

## 2 Experimental Design, Materials and Methods

The data were collected as part of CIMMYT's knowledge hub in Chiapas, Mexico. Crop production in this hub is characterized by rainfed maize farming systems with a mix of small-scale low-input self-consumption farmers, referred to as the Landrace-based system and medium-scale medium-input mechanized semi-commercial farmers selling to local markets, referred to as the Hybrid system.

The hubs comprise different levels of agronomic experimentation: research platforms are typical controlled experiments; demonstration modules are on farmers' land and involve side-by-side fields comparisons of different technologies and management practices; extension areas are fields where farmers have implemented management changes after testing them in demonstration modules; impact areas are other fields where farmers adopted innovations without being directly connected to the hub [2]. The data in this dataset was collected in modules and extension areas.

Farm advisors recorded the data on the identification of each field, maize yield and field management over seven growing seasons (2012-2018) during regular field visits. Farmers’ data were captured in CIMMYT-developed field books using an in-house developed system (Bitácora electrónica MasAgro, BEM) which allowed for logic, entry constraints (i.e. ranges in the answers-input) sub-structure repetitions and geo-referenced information. Data collectors could work online and offline in the field, save submissions at any point and send them to CIMMYT servers. Each observation represents one crop event with a set of correspondent practices in one parcel of land, usually a small field (< 5 ha) or part of the field. Identification of the field included an automatically generated unique ID, geolocation of the field (latitude and longitude determined by at least 7 decimals) and elevation. Farm advisors recorded field management data obtained from farmers that included cultivar planted, tillage practice used, planting date and fertilization dose and timing (dataset contains N, P_2_O_5_, K_2_O dose). Grain yield was determined by harvesting an area of 5 m by 1.5 m (two rows of maize wide) in five representative points of each plot. Moisture content of the grain was determined and adjusted to 14% for reporting. In the case of subsistence farmers, located mainly in the Ocosingo and Los Altos regions of Chiapas, yield in the extension areas was estimated by the farmers, because harvest was performed over the course of several months. Average grain yield per municipality was obtained from the surveys published by SIAP [3].

The data was filtered to remove points with coordinates outside the state's boundaries, the observations with missing data for the variables used, and some extreme values. Data wrangling was performed using the R software version 4.1 [4] and the package `dplyr' [5]. Spatial blurring was conducted (reduced to 2 decimals) to avoid identifying farmers’ fields.

Soil information was obtained from a polygon map of soil units with functional properties constructed using soil samples from Mexico's National Institute of Statistics and Geography (INEGI) open-access datasets [6]. The point coordinates from the field observations were spatially overlaid in the soil map polygons using the R software package "sf" [7]. The soil attributes clay, cation exchange capacity (CEC), soil organic matter (SOM), and pH were extracted. Elevation and slope were derived from SRTM digital elevation data [8].

The weather dataset was assembled using Daymet gridded daily surface weather data with 1 km spatial resolution [9]. The point coordinates from the field observations were spatially overlaid in the Daymet gridded data using the R software package "raster" [10]. The values corresponding to each pixel for precipitation, solar radiation, maximum temperature, minimum temperature, and vapor pressure were extracted. The daily data were aggregated into ten-day intervals to reduce the number of features used in the model. The weather data were organized according to the planting dates, starting 60 days before planting and running up to 240 days after planting, thus creating 30 new features for each variable, extending beyond the growing season and not representing only the conditions in which plants were growing.

## Ethics Statements

This work involved human subjects, to the extent that the data collected included spatial location of farmers’ fields, names, individual production costs and income information related to agronomic activities. Therefore, a relevant informed consent was obtained by extension agents through the data collection system, describing the use of the data, as well as the sharing code for third parties.

Farmers agreed to provide information with which CIMMYT could (a) generate and disseminate statistical information related to any of the areas of scientific research in which CIMMYT specializes, after anonymizing the data obtained; (b) provide information on courses and training that may be of interest to the owner of the data; (c) generate information and/or studies regarding agriculture or related disciplines, including uses and practices in agriculture, socioeconomic conditions in certain populations/zones/regions. The Data referred to in sections (a), (b) and (c) may be transferred, shared and/or assigned to authorities that have the powers or the right to access them.

## CRediT Author Statement

**Rodrigo G. Trevisan:** Conceptualization, Formal analysis, Investigation, Writing – original draft, Visualization; **Nicolas. F. Martin:** Supervision, Funding acquisition, Conceptualization, Writing – review & editing; **Simon Fonteyne:** Investigation, Writing – review & editing; **Nele Verhulst:** Investigation, Writing – review & editing; **Hugo A. Dorado Betancourt:** Investigation, Writing – review & editing; **Daniel Jimenez:** Funding acquisition, Writing – review & editing; **Andrea Gardeazabal:** Project administration, Investigation, Data curation, Writing – review & editing.

## Declaration of Competing Interest

The authors declare that they have no known competing financial interests or personal relationships that could have appeared to influence the work reported in this paper.
